# Site-Directed Mutagenesis Increased the Catalytic Activity and Stability of *Oenococcus oeni* β-Glucosidase: Characterization of Enzymatic Properties and Exploration of Mechanisms

**DOI:** 10.3390/ijms26093983

**Published:** 2025-04-23

**Authors:** Junpeng Zuo, Jie Zhang, Hongyu Ma, Yanqi Zhang, Pengyan Li, Ying Wu, Pingping Tian, Qiuxia Fan, Li Cao, Jianrui Sun, Shaobin Gu

**Affiliations:** 1 College of Food and Bioengineering, Henan University of Science and Technology, Luoyang 471023, China; 18303631677@163.com (J.Z.); 18240538532@163.com (H.M.); 13345296887@163.com (Y.Z.); superlipengyan@163.com (P.L.); wuying2000@126.com (Y.W.); tp2021@haust.edu.cn (P.T.); 9906488@haust.edu.cn (Q.F.); xbcaoli@163.com (L.C.); dasheng@haust.edu.cn (J.S.); 2 Henan Engineering Research Center of Food Microbiology, Luoyang 471023, China

**Keywords:** β-glucosidase, catalytic activity, thermal stability, site-directed mutation, molecular docking

## Abstract

*Oenococcus oeni* β-glucosidase can significantly improve food flavor, but its catalytic activity and stability performance need to be enhanced. In this study, the two dominant mutants III and IV were obtained by site-directed mutation of key amino acid residues in the catalytic pocket of *Oenococcus oeni* β-glucosidase. Compared with the wild-type enzyme, the activities of mutants III and IV were increased by 2.81 and 3.18 times, respectively. Their thermal stability was also significantly improved. Mutants III and IV showed a significant increase in affinity for the standard substrate *p*-NPG, with the *K*_m_ value decreasing by 18.2% and 33.3%, respectively. Molecular docking analysis indicated that hydrogen bonding and π–π aromatic interactions were the primary factors influencing the changes in enzyme properties, with F133 and N181 identified as key amino acids affecting catalytic activity and stability. This research is of great significance for enhancing food flavor and expands the potential industrial applications of *Oenococcus oeni* β-glucosidase.

## 1. Introduction

β-glucosidase (BGL, EC3.2.1.21) is also known as β-D-glucoside glucose hydrolase, gentiobiase, cellobiase and amygdalinase. It is an important component of the cellulase system. The main function of BGLs is to catalyze the hydrolysis of β-1,4-glycosidic bonds at the non-reducing ends of alkyl and aryl groups and different oligosaccharides, releasing β-D-glucose and its corresponding ligands [1]. In addition, BGLs also have a certain hydrolysis activity on β-1,1, β-1,2, β-1,3 and β-1,6 glycosidic bonds. BGLs can also weakly hydrolyze β-D-cellulosic glycosides, β-D-galactoside, β-D-hydroxyglucoside and α-D-glucoside bonds, releasing a variety of ligands [2]. The sources of BGLs are diverse and can be generally divided into plant sources, animal sources and microbial sources [3]. In recent years, due to the advantages of their high yield, low price and high catalytic efficiency, the current research has focused on BGLs derived from microbiology. Among them, BGLs derived from fungi such as *Aspergillus niger* and yeast were the research hotspots, while the BGLs from *Oenococcus oeni* were rarely reported [4].

BGLs are widely used in the bioenergy, chemical and food industries due to a variety of functions such as their glycoside hydrolysis ability and substrate specificity [5]. In the food industry, β-glucosidase can hydrolyze hundreds of β-glucoside flavor precursors, which can significantly improve food flavor and quality [6]. For example, soybean contains 12 different isoflavones. Endogenous β-glucosidase can hydrolyze isoflavones to obtain β-glucoside ligands, such as phenolic aglycones [7]. Many other related studies have shown that β-glucosidases can improve the aroma quality of tea by increasing the contents of aromatic alcohols and their oxides, such as nerol, geraniol and phenylethanol. They also have a positive effect on the contents of some aliphatic compounds, terpenes and aromatic compounds [8,9]. In the wine industry, adding β-glucosidase can significantly enhance wine aroma, directly manifested as increasing the contents of terpenes in wine [10]. In addition, it can also reduce the degradation rate of anthocyanins in wine, which is conducive to wine fermentation [11,12].

Due to the high industrial application value of BGLs, designing and producing BGLs with higher catalytic activity and stability through various methods has always been a hot research direction. Many studies have also been conducted to improve the enzymatic properties of existing BGLs. Molecular docking technology has been widely used to construct enzyme–substrate complex models for predicting binding modes and catalytic mechanisms [13]. Xia et al. obtained a BGL3A mutant with increased catalytic activity through molecular docking and saturation mutagenesis [14]. Binding energy scanning is also a commonly used method to guide the molecular modification of BGLs. It can predict the changes of binding energy in advance, making the mutation selection more reasonable, and successfully realize the activity modification of various industrial enzymes [13]. Teze et al. obtained a mutant with an increased yield, from 36% to 90%, by mutating the −1 subsite of BGLs in *Thermus thermophilus* by binding energy scanning. It was concluded that decreasing the interaction of the glycosyl subsite decreases the stability of the transition state in the reaction. This has a greater effect on the hydrolysis reaction than on the transglycosylation reaction, thus increasing the transglycosylation/hydrolysis ratio [15]. Hassan et al. also modified the β-glucosidase in *Halothermothrix orenii* by rational design. They constructed five mutants in the vicinity of the −1 and +1 subsites. One of the mutants near the −1 subsite synthesized oligogalactose in an increased yield of 57.4% [16]. Yang et al. also demonstrated similar results using β-glucosidase from *Thermotoga naphthophila*, with the most effective mutant exhibiting a significant increase in hydrophilicity of the -1 subunit [17]. Lee et al. improved the thermal stability of BGLs derived from *Trichoderma reesei* by altering the amino acids that are external to the active site [18]. The study by Ranaei et al. showed an increase in the enzyme’s affinity to the substrate by decreasing the glycosylation site, increasing the catalytic efficiency to 143% of the wild-type enzyme [19].

*Oenococcus oeni* is one of the commonly used bacterial strains in wine fermentation processes. The β-glucosidase produced by *Oenococcus oeni* can effectively act on the glycosidically bound aroma precursors in the wine matrix, promoting the cleavage of the bound aroma molecules and releasing a large amount of free volatile aroma compounds, thereby achieving a dual enhancement in both the complexity and intensity of the aroma in the wine fermentation system. Consequently, the aim of this study was to enhance the catalytic performance of *Oenococcus oeni* β-glucosidase through molecular modification techniques. Based on the significant application value and our previous systematic research on *Oenococcus oeni* β-glucosidase, in this study, we used a site-directed mutagenesis strategy to modify the *Oenococcus oeni* β-glucosidase to obtain mutants with high enzyme activity and good stability. Additionally, further expression, purification and enzymatic characterization were carried out on the mutants. This study is expected to provide theoretical guidance for the modification of BGLs to broaden their application fields.

## 2. Results and Discussion

### 2.1. Screening of Mutation Sites and Mutants

**Figure 1:**
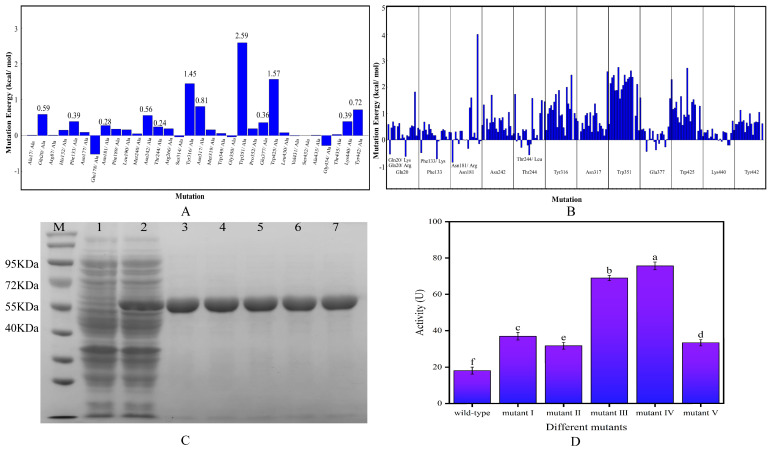
(**A**) Results of alanine scan; (**B**) results of single mutation binding energy change; (**C**) SDS-PAGE analysis (M: marker; 1: empty vector control; 2: bacteriophage containing the expression plasmid; 3–7: target protein purification of mutants I to V); (**D**) determination of enzyme activity (Lowercase letters indicate significant differences in catalytic activity between different mutants and wild type, *p* < 0.05).

The results of alanine scanning and single mutation binding energy changes showed that 12 residues (Trp351, Trp425, Tyr316, Asn317, Tyr442, Gln20, Asn242, Phe133, Lys440, Glu377, Asn181 and Thr244) had a mutation energy greater than 0.2 kcal/mol, indicating that these residues were key residues in the structure of β-glucosidase (Figure 1A). Four of the 12 key residues can form hydrophobic interactions with small molecules, and two can form hydrogen bonds, which also proves the rationality of alanine scanning. Single mutations were performed on the above 12 residues. The mutation results are shown in Figure 1B. A single point mutation was identified for those with a binding energy change of less than −0.5 kcal/mol, leading to the identification of the mutation sites Q20K, Q20R, F133K, N181R and T244L, which were designated as mutant I, mutant II, mutant III, mutant IV and mutant V, respectively.

It can be seen in Figure 1C that the expression of the bacterium’s own proteins were high when the target gene was not introduced (lane 1), and after the insertion of the target gene (lane 2), there was a clear band at 55 KDa with a higher expression level, indicating the successful expression of the target protein. Lanes 3–7 were the results of protein purification of the five mutants, which had only a single and cleared band at 55 KDa, and there is no other heterozygous band, indicating that the target proteins have been well purified, and the molecular weight of 55 KDa was consistent with the prediction. It can be seen from Figure 1D that the enzyme activities of mutants I–V were all greater than that of the wild-type enzyme, among which the enzyme activity of mutant III and mutant IV increased more significantly, reaching 3.81 and 4.18 times that of the wild-type enzyme, respectively. Consequently, these two mutants were chosen for further experiments in this study.

### 2.2. Characterization of Enzymatic Properties

#### 2.2.1. Effects of Temperature on Enzyme Activity

**Figure 2:**
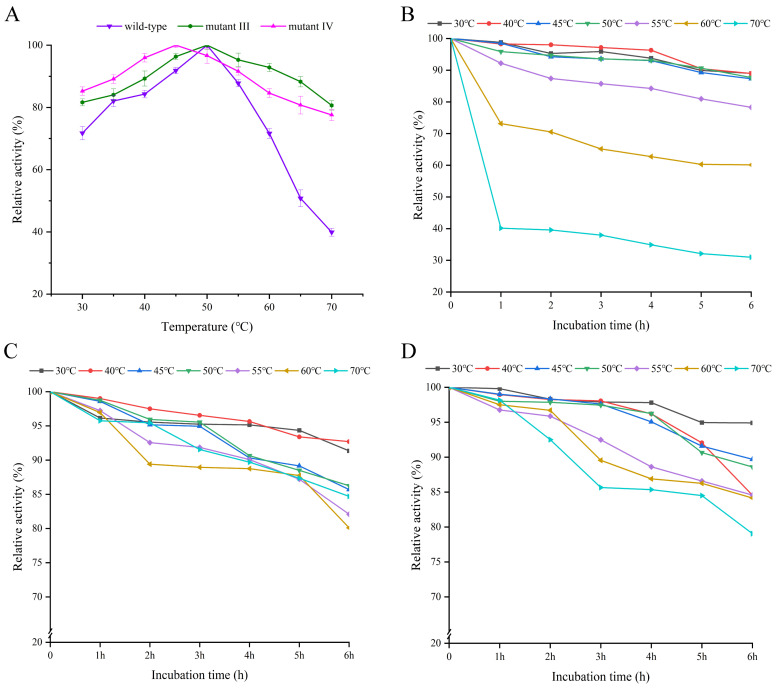
The optimum temperature and thermal stability of the wild-type enzyme and mutants III/IV. (**A**) Optimum temperature of wild-type enzyme and mutants III/IV; (**B**) thermal stability of the wild-type enzyme; (**C**) thermal stability of mutant III; (**D**) thermal stability of mutant IV. The optimum temperature was obtained by controlling the same conditions of pH.

Temperature is one of the main factors affecting enzyme activity, and the thermal stability of enzymes directly reflects their adaptability and application potential to various environmental conditions [20]. As shown in Figure 2A, the optimal catalytic temperature for both the wild-type enzyme and mutant III was 50 °C, while that for mutant IV was 45 °C. For the wild-type enzyme, the catalytic activity was maintained at a high level when the temperature was between 35 and 55 °C, with the relative activity exceeding 80%. However, its relative activity decreased sharply to below 50% when the temperature exceeded 65 °C. For mutants III/IV, their relative activity remained above 80% at 65 °C, demonstrating stronger heat resistance. Zhang et al. mutated *Leuconostoc mesenteroides* dextransucrase, in which the optimal temperature of mutants L838C–V887C and A948C–A1013C were increased by 10 °C compared to the wild-type enzyme [21]. Additionally, the mutants III/IV also demonstrated more excellent thermal stability. As shown in Figure 2B, when the wild-type enzyme was incubated below 50 °C, the enzyme activity could be maintained at a high level, but when the temperature exceeded 50 °C, its activity decreased significantly with the increase of incubation time. In contrast, the catalytic activities of mutants III/IV remained above 80% after incubation at 70 °C for six hours (Figure 2C,D), indicating a significant improvement in the thermal stability of the enzyme after mutation, which provided a possibility for its application under high temperature conditions. Dawei et al. applied combinatorial targeted mutagenesis to inulosucrase from *Lactobacillus casei* UMB7800, resulting in three mutants, G531P–S344D, G531P–H434P and G531P–E256D, with significantly enhancement in thermal stability [22]. Additionally, the half-life of the wild-type enzyme and mutants at their optimal temperatures are shown in Table S2. The half-life of the wild-type enzyme was 23 h, and the half-lives of mutant III and mutant IV were prolonged compared with those of the wild-type enzyme by 3 h and 8 h, respectively. The prolongation of the half-life will well broaden the application field of mutants.

#### 2.2.2. Effects of pH on Enzyme Activity

**Figure 3:**
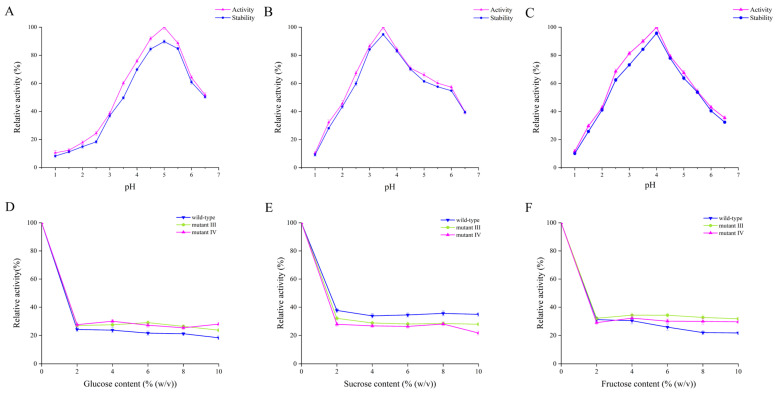
(**A**) The optimum pH and acid stability of the wild-type enzyme; (**B**) the optimum pH and acid stability of mutant III; (**C**) the optimum pH and acid stability of mutant IV; (**D**) effect of glucose on enzyme activity; (**E**) effect of sucrose on enzyme activity; (**F**) effect of fructose on enzyme activity. The optimum pH was obtained at the optimum temperature for both the wild-type enzyme and mutants. The results of the effects of different sugars on enzyme activity were obtained under the optimum temperature and optimum pH for both the wild-type enzyme and mutants.

The optimal reaction pH and acid stability of the wild-type enzyme and mutants III/IV were determined. The results are shown in Figure 3A–C. The optimal pHs for the wild-type enzyme and mutants III/IV were 5.0, 3.5 and 4.0, respectively, which indicated that the mutants had higher catalytic activity than the wild-type enzyme under more acidic conditions. It is worth mentioning that when the pH was 3.0, the relative activity of the wild-type enzyme was only 38.54%, while the relative activity of the mutant enzymes exceeded 60%, especially mutant III, whose relative enzyme activity even reached 86.54%. Nielsen et al. replaced phenylalanine (Phe) with alanine (Ala) at position 290 of α-glucosidase from *Chimeric Bacillus* Ba2. Since the side chain of Phe has a larger size and a different charge distribution compared to that of Ala, this structural change may affect the electrostatic interactions near the enzyme’s active center, which in turn changes the enzyme’s pH activity distribution [23]. Shi et al. performed a targeted mutation of GadB1 in *Lactobacillus brevis* Lb85 and obtained mutant T17I-D294G-Q346H and mutant E312S, and the combination of these two mutations altered the optimal pH of mutant T17I-D294G-Q346H-E312S compared to the wild-type enzyme [24]. Additionally, the trends of the curves in Figure 3A–C were largely similar. After being incubated in various pH buffers for 12 h, the catalytic activities of the three BGLs remained close to their initial values, indicating that all three enzymes exhibited good acid stability.

#### 2.2.3. Effects of Different Sugars on Enzyme Activity

As the enzymatic products of BGLs, glucose and its analogues can inhibit the activity of BGLs to varying degrees [25]. Consequently, the impact of three sugars (glucose, sucrose and fructose) on enzyme activity was examined. As shown in Figure 3D–F, all three sugars had inhibitory effects on enzyme activity. Specifically, the relative activities of mutants III/IV at different glucose concentrations were essentially the same, whereas the relative activity of the wild-type enzyme was lower than that of the mutants. With the increase of glucose concentration, the activity of all three enzymes significantly decreased. When the glucose concentration was 10%, the enzyme activities of the wild-type and mutants III/IV were maintained at 18.35%, 23.75% and 28.03%, respectively. It has been reported that the relative binding position of glucose within the channels of the catalytically active center of BGL is a key reason for the regulation of glucose tolerance. When glucose is present in the reaction environment, glucose competes with substrate molecules for the catalytic activity center of BGL, resulting in a decrease in the enzymatic activity of BGL. Compared with the wild-type, the mutants have a deeper and narrower substrate-binding channel that prevents glucose from entering the catalytically active center of the enzyme [26], so the mutants III/IV have higher enzyme activity and higher glucose tolerance than the wild-type enzyme. It has also been reported that the hydrophobicity and spatial position of the channel entrance at the active site also play an important role in glucose tolerance [27]. Sun et al. mutated the phenylalanine residue at position 171 in BGLA to a tryptophan residue, resulting in BGLA (F171W) exhibiting higher glucose tolerance than wild-type BGLA [28]. When sucrose was introduced into the reaction environment, the relative activity of the three enzymes decreased significantly with the increase of sucrose concentration. When the sucrose concentration was increased to 10%, the enzyme activity of the wild-type enzyme and mutants III/IV remained at 35%, 30% and 21.71%, respectively. When the fructose concentration increased from 0 to 10%, the relative activity of the wild-type enzyme showed a downward trend, and the relative activity decreased to 28.75%. However, the relative activity of mutants III/IV firstly showed a slight increase when the fructose concentration rose from 2% to 4%, and then decreased. When the fructose concentration was at 10%, the relative enzyme activity of mutants III/IV (31.82% and 29.68%, respectively) was still higher than that of the wild-type enzyme. Although glucose, sucrose and fructose belong to different types of sugar categories, their structures exhibit certain similarities. Like glucose, when sucrose and fructose are present in the environment, they can compete with substrate molecules for the active site of β-glucosidase, thereby exerting an inhibitory effect on the catalytic activity of β-glucosidase. In summary, the wild-type enzyme demonstrated better tolerance to sucrose compared to mutants III/IV, while mutants III/IV exhibited greater tolerance to glucose and fructose than the wild-type enzyme.

#### 2.2.4. Effects of Metal Ions and Some Additives on Enzyme Activity

**Figure 4:**
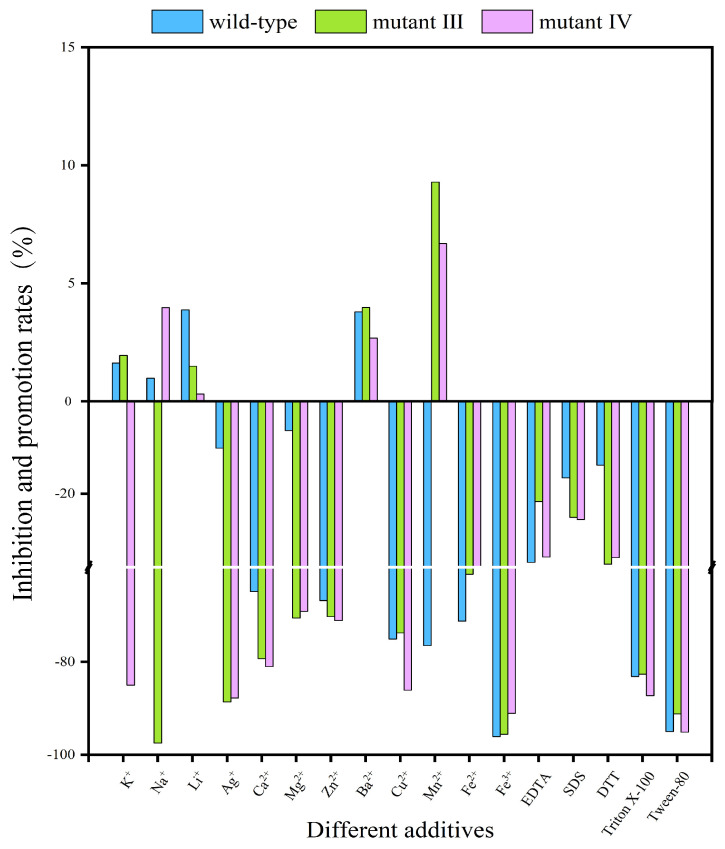
The effects of metal ions and some additives on the activity of the wild-type enzyme and mutants III/IV. The results were obtained under the conditions of the optimum temperature and optimum pH for both the wild-type enzyme and mutants.

In the application process of BGLs, the presence of metal ions and some additives will greatly affect the catalytic activity of the enzyme. These metal ions and additives have a wide range of sources, such as soil, water and fertilizers [29]. Figure 4 and Table 1 showed the effects of different metal ions and additives on enzyme activity, and their semi-inhibitory concentration (IC_50_). As depicted in Figure 4, at a concentration of 5 mM, K^+^ enhanced the enzyme activity of both the wild-type enzyme and mutant III, but strongly inhibited mutant IV, with an inhibition rate of 85.02%. Na^+^ increased the activity of the wild-type enzyme and mutant IV, while inhibiting mutant III at a rate of 97.45%. Mn^2+^ inhibited the wild-type enzyme’s activity by 76.51%, but promoted the activity of mutants III and IV. Additionally, Li^+^ and Ba^2+^ were the only metal ions that enhanced the activity of both the wild-type enzyme and mutants III and IV. As for other metal ions, such as Ag^+^, Ca^2+^, Mg^2+^, Zn^2+^, Cu^2+^, Fe^2+^ and Fe^3+^, they all had inhibitory effects on the enzyme activity of the three BGLs. Various additives such as EDTA, SDS, DTT, Triton X-100 and Tween-80 also inhibited catalytic activity to different extents, with Tween-80 exhibiting the strongest inhibition and SDS the weakest. The SDS molecule contains ROSO_3_^−^, which has high electronegativity and can interact with the hydrophobic cavity formed by amino acid residues, creating competitive antagonism with the substrate and significantly reducing BGL catalytic activity. In contrast, Triton X-100 has a -(CH2-CH2-O)_n_-hydrophobic chain that can also bind to the hydrophobic cavities and compete with the substrate, leading to decreased catalytic activity. Tween-80 and DTT, as a non-ionic surfactant and metal ion protease inhibitor, can effectively inhibit the formation of disulfide bonds on protein surfaces, thereby reducing enzyme activity. Furthermore, Tween-80 can disrupt intramolecular and intermolecular hydrogen bonds, causing protein unfolding and altering its secondary and tertiary structures, ultimately inhibiting enzyme activity.

#### 2.2.5. Analysis of Enzymatic Reaction Kinetic Constants and Substrate Specificity

The kinetic parameters of the wild-type enzyme and its mutants catalyzing on four kinds of substrates are shown in Table 2. Overall, all three BGLs were able to catalyze these substrates, but the kinetic parameters of the reactions were obviously different. The parameter *V*_max_ reflects the maximum reaction rate when the enzyme catalyzes the substrate, and the *V*_max_ of *p*NP-β-glu catalyzed by the three BGLs was greater than those of the other three kinds of substrates. The *V*_max_ of the mutants III/IV in catalyzing three substrates (*p*NP-β-glu, *p*NP-β-gal and *p*NP-β-xyl) were all larger than that of the wild-type enzyme, indicating that the mutants had a higher catalytic efficiency. *K*_m_ represents the affinity between enzymes and substrates, i.e., the smaller the *K*_m_ value, the greater the affinity. In this study, the three enzymes had the highest affinity with substrate *p*NP-β-glu, with *K*_m_ values all less than 0.33 ± 0.03 mM. Additionally, the three enzymes had the lowest affinity with substrate *p*NP-β-xyl, with the highest *K*_m_ value reaching 1.31 ± 0.22 mM. In addition, mutations increased the affinity of the enzyme to the substrates *p*NP-β-glu and *p*NP-β-xyl, while decreasing the affinity to substrate *p*NP-α-glu. Similar findings were reported by Tonin et al., who noted that targeted mutations in hydroxysteroid dehydrogenases led to decreased substrate affinity in some mutants [30], aligning with our results. The specificity constant, *K*_cat_/*K*_m_, helps identify the most suitable substrate when an enzyme can catalyze multiple substrates. As shown in Table 2, the *K*_cat_/*K*_m_ value for *p*NP-β-glu was the highest for both the wild-type enzyme and mutants III/IV, indicating that *p*NP-β-glu is the most suitable substrate for all three enzymes.

### 2.3. Mechanism Analysis of Enzymatic Property Change

Some special sites in the amino acid structure of the enzyme are the key to its high catalytic activity, such as hydrogen bonding [31], hydrophobic bonding [32], salt bridges [33], aromatic–aromatic, cation–aromatic [34] and other non-covalent and disulfide bridges connecting peptide chains [35]. It is essential to deeply analyze the connection between enzyme activity and structure to optimize enzyme function. In this research, we initially predicted the isoelectric points, molecular weights, instability coefficients and other characteristics of the three BGLs. The findings (Table S3) indicated that there were minor differences in the pI and molecular weight among the three BGLs. All three were identified as stable proteins (with stability factors below 40), with mutant IV exhibiting the highest stability, followed by mutant III. In contrast, the wild-type enzyme demonstrated the lowest stability compared to the mutants.

**Figure 5:**
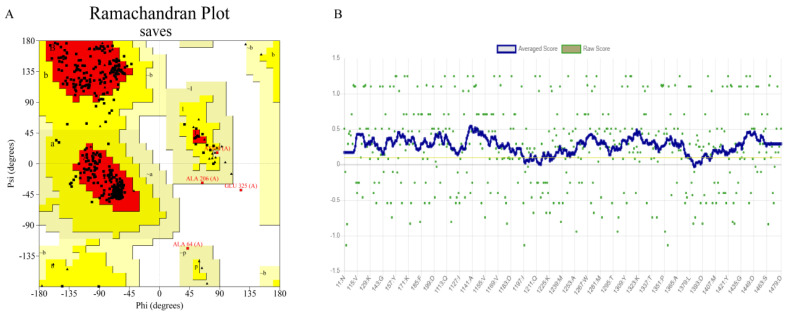
Quality evaluation of the 3D model of the wild-type enzyme. (**A**) Ramachandran plot (Black represents amino acid residues, red represents the core region, yellow represents the allowed region, and white represents the not allowed region. Residues in most favoured regions (A, B, L), Residues in additional allowed regions (a, b, l, p), Residues in generously allowed regions (~a, ~b, ~l, ~p)); (**B**) Verify 3D score (Yellow lines represent 0.1 standard lines).

A search of the PDB database revealed that the crystal structure of β-glucosidase from *Lactobacillus plantarum* (ID: 3QOM) showed a high degree of sequence identity (51.00%) with the wild-type enzyme. Therefore, the crystal structure of 3QOM is a good template for constructing a 3D structural model of the wild-type enzyme. The results of the homology modeling and the reasonableness assessment of the model are shown in the form of a “Ramachandran plot” (Figure 5A). The amino acid residues located in the stereochemically permissive region reached 99.80%, indicating that 99.80% of the amino acid residues in the three-dimensional structure of the wild-type enzyme had a spatial conformation within a reasonable range, which was in accordance with the energy law of stereochemistry. The compatibility between the 3D structural model of the wild-type enzyme and its own amino acid sequence was assessed by the Verify 3D program (https://www.doe-mbi.ucla.edu/verify3d/, (accessed on 3 November 2024)). As shown in Figure 5B, the percentage of amino acid residues with scores above 0.1 was 92.07%, which was in line with the requirements of the evaluation program. Taken together, the 3D structural model of the wild-type enzyme was highly reliable and stable, and can be used in the subsequent molecular docking process.

The number of salt bridges between the wild-type enzyme and mutants before and after binding to the substrate were analyzed using Protein Tools (www.proteintools.uni-bayreuth.de, (accessed on 3 April 2025)), and the results are shown in Table 3. The number of salt bridges of the wild-type enzyme and mutant III/IV were 22, 18 and 18, respectively, and the number of salt bridges after binding to the substrate *p*-NPG remained the same, but mutant III/IV had one salt bridge connected to the substrate *p*-NPG, which may make mutant III/IV have an increased affinity with the substrate *p*-NPG, thus enhancing their catalytic activity [33].

**Figure 6:**
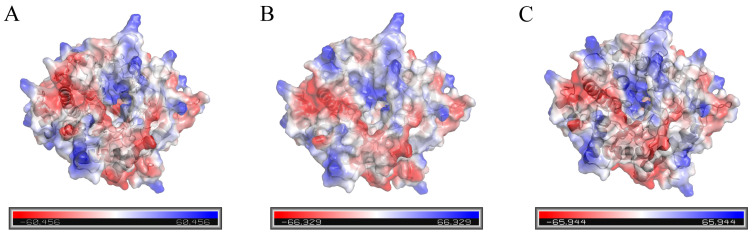
Analysis of the surface electrostatic potential of the wild-type enzyme and mutants. ((**A**) Wild-type enzyme; (**B**) mutant III; (**C**) mutant IV).

The surface electrostatic potential of the wild-type enzyme and mutants were analyzed by PyMOL, and the results are shown in Figure 6. The Phe133 and Asn181 of the wild-type enzyme were replaced by the high-potential amino acids Lys133 and Arg181, respectively. The surface electrostatic potential of mutant III/IV changed and was significantly higher than that of the wild-type. Studies have shown that the change of the electrostatic potential on the surface of the protease will change the stability of the enzyme [36], and the mutation leads to the increase of the electrostatic charge on the surface of the β-glucosidase, which makes the surface of the β-glucosidase protein easier to form a hydrated film and increases the solubility of the β-glucosidase. The rigidity of the C-terminal and N-terminal structures of the mutants has also been significantly enhanced, thereby delaying the expansion of the flexible region and improving the stability of the mutants III/IV [37].

**Figure 7:**
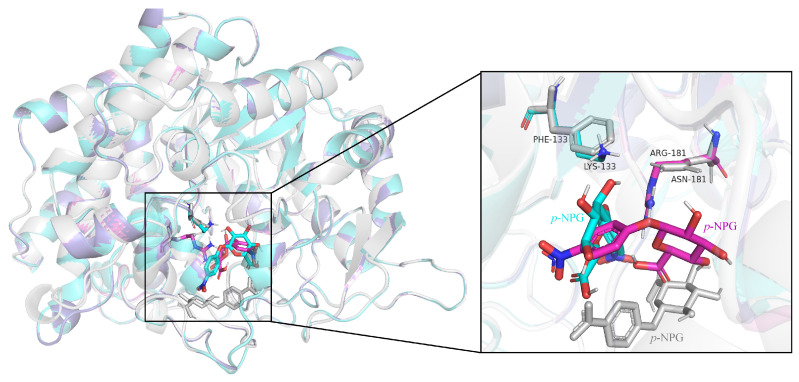
Comparative effect of superposition of substrate binding sites in the wild-type enzyme and mutant active sites (gray: wild-type enzyme; cyan: mutant III; purple: mutant IV).

It can be seen from Figure 7 that, compared with the wild-type enzyme, after the mutant III/IV binds to the substrate *p*-NPG, *p*-NPG can penetrate deeper into the center of the active pocket and enhance the affinity of the mutants with the substrate.

**Figure 8:**
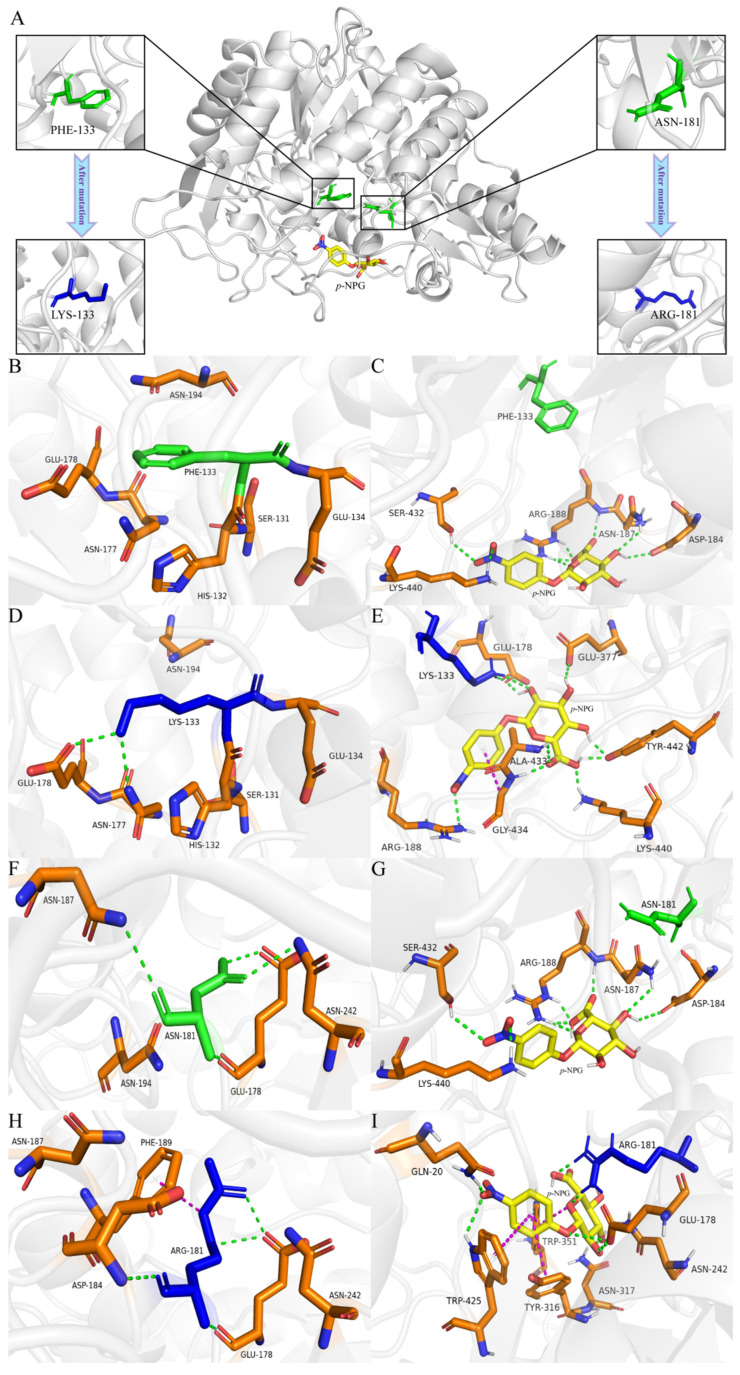
(**A**) The 3D protein model and mutation site information of the wild-type enzyme; (**B**) hydrogen bonding interactions between the F133 site and its surrounding amino acids in the wild-type enzyme; (**C**) hydrogen bonding interactions between *p*-NPG and the surrounding amino acid residues after molecular docking between the wild-type enzyme and *p*-NPG; (**D**) hydrogen bonding interaction between the K133 site and the surrounding amino acids of mutant III; (**E**) hydrogen bonding and π–π interactions between *p*-NPG and the surrounding amino acid residues after molecular docking between mutant III and *p*-NPG; (**F**) hydrogen bonding interaction between the N181 site and the surrounding amino acids of the wild-type enzyme; (**G**) hydrogen bonding interactions between *p*-NPG and the surrounding amino acid residues after molecular docking between the wild-type enzyme and *p*-NPG; (**H**) hydrogen bonding and π–π interaction between the R181 site and the surrounding amino acids of mutant IV; (**I**) hydrogen bonding and π–π interactions between *p*-NPG and the surrounding amino acid residues after molecular docking between mutant IV and *p*-NPG. (The green dashed line represents a hydrogen bond, and the purple dashed line represents a π–π interaction).

To thoroughly investigate the mechanisms behind the enhancement of enzyme activity and stability, we analyzed the alterations in the microenvironment surrounding the mutation site through molecular simulations. The results are shown in Figure 8. The distances of the mutated amino acid residues from the surrounding critical amino acid residues of the wild-type and mutants were measured using PyMOL. For the wild-type enzyme, the distances of F133 from the critical amino acids S131, H132, E134, N177, E178 and N194 are 6.3 Å, 5.0 Å, 4.4 Å, 7.3 Å, 8.9 Å and 5.8 Å, respectively, and the distances between N181 and the key amino acids E178, N187, N194 and N242 are 5.6 Å, 7.4 Å, 5.8 Å and 5.4 Å, respectively. The distances between K133 of mutant III and the key amino acids S131, H132, E134, N177, E178 and N194 are 5.0 Å, 5.0 Å, 4.4 Å, 7.3 Å and 8.9 Å, respectively, and the distances of R181 of mutant IV from the key amino acids E178, D184, N187, F189 and N242 are 3.6 Å, 5.1 Å, 7.2 Å, 3.9 Å and 4.5 Å, respectively. Compared to the wild-type enzyme, the mutated LYS133 and ARG181 showed reduced distances to key amino acids. It can be seen from Figure 8A that F133 and N181 of the wild-type enzyme are located in the center of the catalytic active pocket, indicating that the two amino acids are important for the wild-type enzyme to exert catalytic activity. The substrate molecule *p*-NPG is located in the edge region of the active pocket. The F133 of the wild-type BGL (Figure 8B) and its surrounding amino acids rely on van der Waals forces to maintain the spatial structure without hydrogen bonding. The F133 contains a benzene ring, which may lead to steric hindrance and exclude *p*-NPG from the catalytic activity pocket, thereby affecting the activity of BGL [14]. Mutations may lead to changes in the ionization state and charge state of amino acid residues, which in turn affect the interaction with other amino acid residues and the substrate, thereby affecting the conformational stability, structural stability and catalytic activity [38]. When the substrate *p*-NPG was present (Figure 8C), it formed eight hydrogen bonds with D184, N187, R188, S432 and K440 in the edge region of the catalytically active pocket, and did not form hydrogen bonds with F133. After the non-polar and hydrophobic F133 was mutated to the polar and hydrophilic K133 (Figure 8D), K133 formed a hydrogen bond with its surrounding amino acid residue E178. This change in polarity and hydrophobicity enhances the hydrophilicity at the center of the catalytic pocket, facilitating substrate binding and product release [39]. The increased polarity of the amino acids boosts the affinity between BGL and *p*-NPG, allowing the substrate to penetrate further into the catalytic pocket and achieve better geometric compatibility with BGL. Additionally, the loss of the benzene ring reduces steric hindrance and increases flexibility, further enhancing enzyme catalytic activity [40]. After the mutation, *p*-NPG forms two hydrogen bonds with the crucial amino acid K133 (Figure 8E) and establishes eight hydrogen bonds along with one π–π aromatic interaction with the surrounding residues E178, R188, E377, A433, G434, K440 and Y442. Compared to the wild-type, this results in two additional lattice hydrogen bonds and one π–π aromatic interaction between the enzyme and substrate, which promotes their binding and improves enzyme activity [14].

The N181 of the wild-type enzyme formed four hydrogen bonds with its surrounding amino acid residues E178, N187 and N242 (Figure 8F). In the presence of the substrate *p*-NPG (Figure 8G), it established hydrogen bonds with amino acids in the catalytic activity pocket of the enzyme, but not with N181. After N181 was mutated to R181 (Figure 8H), the side chain of the amino acid became longer, resulting in the formation of four hydrogen bonds with E178 and D184, as well as a π–π aromatic interaction with F189. The *p*-NPG could enter the center of the catalytic activity pocket more deeply, and formed two hydrogen bonds with R181 of the mutant BGL, and formed six hydrogen bonds and four π–π aromatic interactions with Q20, E178, N242, Y316, N317, W351 and W425 around it (Figure 8I). After mutation, the number of π–π aromatic interactions increased by three compared to the wild-type enzyme, while the number of hydrogen bonds remained the same. However, two hydrogen bonds were now directly linked to the crucial amino acid R181 of the mutant BGL, which strengthened the binding affinity of the mutant BGL for the substrate *p*-NPG and improved its catalytic stability [38].

## 3. Materials and Methods

### 3.1. Bacterial Strains and Materials

*Oenococcus oeni* SD-2a was preserved at the college of food and bioengineering, Henan University of Science and Technology. Competent *E. coli* DH5α was purchased from Sangon Biotech (Shanghai, China). *Lactococcus lactis* MG1363 was purchased from Changsha Fenghui Biotechnology (Changsha, China). The pMG36e plasmid was purchased from HonorGene (Changsha, China), and the SpeedyCut enzyme, DNA polymerase and Ni-IDA 6FF Sefinose (TM) Resin Kit were purchased from Sangon Biotech (Shanghai, China). *p*-Nitrophenyl-β-D-glucopyranoside (*p*-NPG/*p*NP-β-glu), *p*-Nitrophenyl-β-D-galactoside (*p*NP-β-gal), *p*-Nitrophenyl-β-D-xylopyranoside (*p*NP-β-xyl) and *p*-Nitrophenyl-α-D-glucopyranoside (*p*NP-α-glu) were purchased from Yuanye Bio-Technology (Shanghai, China), and M17 broth medium was purchased from Aobox Bio-tech (Beijing, China). Chemicals with purity ≥98% were purchased from Solarbio Life-Science (Beijing, China), mainly including sodium chloride (NaCl), sodium carbonate (Na_2_CO_3_), peptone, glucose, potassium dihydrogen phosphate (KH_2_PO_4_), sodium acetate (NaAc), magnesium sulfate heptahydrate (MgSO_4_·7H_2_O), disodium hydrogen phosphate and anhydrous ethanol.

### 3.2. Prediction of Mutation Sites

β-glucosidase BGL0224 (MT330371.1) from *Oenococcus oeni* SD-2a obtained in our previous research was used as the wild-type enzyme [3]. The protein sequence of the wild-type enzyme was entered into the PDB protein database for comparison. Normally, when the sequence identity between the crystal structure and the target protein exceeds 30%, the known crystal structure can be used as a template to construct a 3D structure model of the target protein. Homology modeling was performed using SWISS-MODEL (https://swissmodel.expasy.org/interactive, (accessed on 11 October 2024)), and the quality of the model was evaluated and characterized using a Ramachandran plot (https://saves.mbi.ucla.edu/, (accessed on 4 November 2024)) and the Verify 3D program (https://www.doe-mbi.ucla.edu/verify3d/, (accessed on 3 November 2024)) for subsequent molecular docking. The 3D structure of wild-type BGL was obtained as the receptor structure of molecular docking. The structure of the substrate was constructed and hydrogenated. The structure was optimized using the MOPAC 2016 program to calculate the atomic charge of PM3. Finally, Autodock Tools 1.5.6 was used to deal with the structure of the ligand and receptor, and the docking box was used to wrap the active site. The number of grid points in the XYZ direction was set to 60 × 60 × 60, the grid spacing was 0.375 Å, the number of dockings was set to 100, and the remaining parameters were used as default values. The Calculate Mutation Energy (Binding) module in the Discovery Studio 2019 software was used to scan all the residues of 5 Å near the *p*-NPG binding pocket.

### 3.3. Construction of Recombinant Plasmid pMG36e-0224 and Its Mutants

The gene of BGL0224 was amplified by PCR using the genomic DNA of *Oenococcus oeni* SD-2a as the template. The purified PCR product and linearized plasmid vector were ligated by T4 DNA ligase to connect the BGL0224 gene fragment to the pMG36e linearized plasmid vector [41]. The recombinant plasmid with the correct sequence was named pMG36e-0224. Based on the rapid site-directed mutagenesis strategy, the recombinant plasmid pMG36e-0224 was used as a template for whole plasmid PCR to construct different mutants. The mutation primers were designed by Primer X (https://www.bioinformatics.org/primerx/cgi-bin/DNA_1.cgi, (accessed on 10 August 2024)). The primer sequences are shown in Table S1. The PCR procedure was as follows: pre-denaturation at 95 °C for 10 min, 97 °C denaturation for 10 s, 56–69 °C annealing for 15 s, 70 °C extension for 60 s, for 35 cycles, and finally, 4 °C heat preservation. The accuracy of the mutation results were verified by sequencing. Next, the wild-type and mutant plasmids were electro-transformed into *Lactococcus lactis* MG1363 for expression and purification [42]. The purified wild-type and mutant target proteins were detected by SDS-PAGE.

### 3.4. Determination of Enzyme Activity

The determination of enzyme activity was conducted according to Dong et al., with slight modifications [43]. First, 10 μL BGLs (10 mg/mL) were added to 490 μL 25 mM *p*-Nitrophenyl-β-D-glucopyranoside (*p*-NPG) prepared with 20 mM sodium phosphate buffer (pH 5.0). After incubation at 37 °C for 30 min, 500 μL 1 M Na_2_CO_3_ solution were added to terminate the reaction. The absorbance was measured at 420 nm, and the *p*-Nitrophenol (*p*-NP) released from the system was determined. The concentration of *p*-NP was calculated according to the standard curve. Under the experimental conditions, the amount of 1 μM *p*-NP produced per minute was defined as a unit of BGL enzyme activity (U).

### 3.5. Determination of Optimal Temperature and Thermal Stability

The optimum temperature and temperature stability of the enzyme were determined with reference to Liu et al., with slight modifications [44]. The lyophilized powders of the wild-type and mutant BGLs were dissolved in 20 mM sodium phosphate buffer (pH 5.0) to give a final concentration of 10 mg/mL. Then, 10 μL of enzyme solution were added to 490 μL of 25 mM *p*-NPG substrate solution configured with sodium phosphate buffer and mixed well. The enzyme reaction was carried out at a temperature range of 30–70 °C (with a gradient of 5 °C) for 30 min. After the reaction was completed, 500 μL of 1 M Na_2_CO_3_ solution were added to develop the color, and the absorbance value of the mixture was measured at 420 nm, and the activity of BGL was calculated according to the standard curve. Thermal stability was determined by reacting the above 500 μL of enzyme–substrate mixture at a temperature range of 30–70 °C for 6 h. Then, the activity of BGL was measured at 1 h, 2 h, 3 h, 4 h, 5 h and 6 h, and the rest of the conditions remained unchanged.

### 3.6. Determination of Optimal pH and Acid Stability

The optimum pH and pH stability of the enzyme were determined with reference to Capaldo et al., with slight modifications [4]. The lyophilized powders of the wild-type and mutant BGLs were dissolved in 20 mM sodium phosphate buffer (pH range 1.0–6.5, gradient 0.5) at different pHs to give a final concentration of 10 mg/mL. Then, 10 μL of enzyme solution were added to 490 μL of 25 mM *p*-NPG substrate solution configured with sodium phosphate buffer (pH range 1.0–6.5 with a gradient of 0.5) and mixed well. The enzyme reaction was carried out at the optimal temperature for 30 min, 500 μL of 1 M Na_2_CO_3_ solution were added at the end of the reaction, and the absorbance value of the mixture was measured at 420 nm, and the activity of BGL was calculated according to the standard curve. Acid stability was determined by reacting the above 500 μL of enzyme–substrate mixture for 12 h at 4 °C, and then the activity of BGL was measured. The rest of the conditions were unchanged.

### 3.7. Determination of Different Sugars on Enzyme Activity

The method for determining the effect of different sugars on enzyme activity referred to Zhao et al., with slight modifications [2]. To determine the effects of different types and concentrations of sugars on BGL enzyme activity, glucose, sucrose and fructose were configured into solutions with concentrations of 0–10% (on a 2% gradient) using 20 mM sodium phosphate buffer. The lyophilized powder of the wild-type and mutant BGLs was dissolved in the prepared sodium phosphate buffer with different concentrations of the three sugar solutions, and the final concentration was 10 mg/mL. A *p*-NPG substrate solution (25 mM) was also prepared with the prepared sodium phosphate buffer with different concentrations of the three sugar solutions. Then, 10 μL of enzyme solution were added to 490 μL of substrate solution and mixed evenly. The reaction lasted for 30 min under the optimal reaction conditions of the BGLs. Then, 500 μL 1 M of Na_2_CO_3_ solution were added to terminate the reaction. Next, the absorbance value of the reaction system at 420 nm was measured, and the activity of the BGLs was calculated based on the standard curve.

### 3.8. Determination of Metal Ions and Some Additives on Enzyme Activity

The method for the determination of the effects of different metal ions and additives on enzyme activity was referred to Zhou et al., with slight modifications [45]. To determine the effects of metal ions and some additives on the BGLs’ activity, lyophilized powders of the wild-type and mutant BGLs were formulated into solutions with a final concentration of 10 mg/mL using 20 mM sodium phosphate buffer as well as 25 mM of *p*-NPG substrate solution and different concentrations (0.1 mM, 0.5 mM, 1.0 mM, 5.0 mM and 10 mM) of metal ion solutions (K^+^, Na^+^, Li^+^, Ag^+^, Mg^2+^, Zn^2+^, Ba^2+^, Ca^2+^, Cu^2+^, Mn^2+^, Fe^2+^ and Fe^3+^) and additive solutions (SDS, DTT, EDTA, TritonX-100 and Tween-80). The enzyme solutions (10 μL), *p*-NPG solution (490 μL) and different concentrations of additive solutions (500 μL) were mixed well. The reaction lasted for 30 min under the optimal reaction conditions of the enzyme. Then, 1mL 1 M Na_2_CO_3_ was added to terminate the reaction. The absorbance value of the reaction system was measured at 420 nm and the activity of the BGLs was calculated according to the standard curve. The IC_50_ (half of the maximum inhibitory concentration) of the reagents on BGL activity was calculated using a logistic function with Growth/Sigmoidal-type nonlinear fitting in Origin Pro 8.0.

### 3.9. Enzymatic Reaction Kinetics and Substrate Specificity

The determination of enzymatic reaction kinetics was conducted according to Zhang et al., with slight modifications [3]. Different concentrations (0.1 mM, 0.2 mM, 0.5 mM, 1.0 mM, 2.0 mM, 5.0 mM, 8.0 mM, 10 mM, 12 mM, 15 mM, 20 mM and 25 mM) of *p*-NPG substrate solutions were prepared with 20 mM sodium phosphate buffer. Then, 100 μg of enzyme lyophilized powder were added to 500 μL of *p*-NPG solutions at different concentrations. Next, this was reacted for 0 min, 5 min, 10 min, 15 min, 20 min, 25 min and 30 min under optimal reaction conditions, and at the end of each set of reactions, 500 μL 1 M of Na_2_CO_3_ solution were added to terminate the reaction. The absorbance value of each reaction system was measured at 420 nm and the amount of *p*-NP corresponding to different reaction times at specific substrate concentrations was calculated from the standard curve. The kinetic constants (*V_max_* and *K_m_*) of each reaction system were determined by fitting using the Hill function in the class of Growth/Inverse Curve for nonlinear fitting in the Origin Pro 8.0 software.

### 3.10. Mechanism Analysis

ExPASy (https://web.expasy.org/protparam/, accessed on 22 July 2024) was used to analyze physical and chemical indicators such as the molecular weight and isoelectric point (pI). Homology modeling of the mutant and wild-type BGLs was performed by the online prediction platform SWISS-MODEL (https://swissmodel.expasy.org/interactive, accessed on 11 October 2024). Autodock Tools 1.5.6 was used to dock the wild-type and mutant BGLs with the substrate *p*-NPG, and the parameter settings for the docking process referred to Section 2.2. The PyMOL 3.1.0 software was used for visual analysis.

### 3.11. Statistical Analysis

All experiments were set up in three parallels. The activities of the wild-type enzyme and five mutants were analyzed by ANOVA at the significance level of *p* < 0.05 using the SPSS 26.0 software, and multiple comparisons of the means were performed using Duncan’s method. The kinetic parameters of the enzymatic reactions for the wild-type enzyme and mutants catalyzing four substrates were subjected to independent sample *t*-tests at a significance level of *p* < 0.05, and the data were expressed as mean ± standard deviation. The experimental results were plotted using the Origin 2021 software.

## 4. Conclusions

In this research, the critical amino acids in the catalytic activity pocket of BGL0224 were identified by binding energy prediction, and the dominant mutants III and IV were further obtained by site-directed mutation. Compared with the wild-type enzyme, the enzyme activities of the mutants were significantly increased under optimal reaction conditions, and the affinity for the substrate *p*-NPG was also enhanced. The mutants were more tolerant to low pH environments. In addition, when glucose and fructose, as well as certain metal ions, were present in the environment, the activities of both the wild-type enzyme and the mutants were inhibited. Molecular simulation results showed that the change of enzyme properties was due to the change of enzyme structure after mutation, mainly manifested as the introduction of new hydrogen bonds and π–π bonds at the active site. This allows the substrate *p*-NPG to function deeper in the catalytic activity pocket, thus enhancing the binding ability between the enzyme and substrate, resulting in higher catalytic activity and stability exhibited by the mutant. Overall, this study expands the industrial applicability of *Oenococcus oeni* β-glucosidase.

## Figures and Tables

**Table 1:** IC_50_ of different metal ions and additives on the wild-type enzyme and mutants.

Additives	Concentration (mM)	Wild-Type Enzyme	Mutant III	Mutant IV
		IC_50_ (mM)	IC_50_ (mM)	IC_50_ (mM)
K^+^	5	Activator	Activator	2.42
Na^+^	5	Activator	0.929	Activator
Li^+^	5	Activator	Activator	Activator
Ag^+^	5	7.43	3.03	2.93
Ca^2+^	5	4.09	3.38	3.28
Mg^2+^	5	8.92	3.37	5.35
Zn^2+^	5	3.87	3.19	2.94
Ba^2+^	5	Activator	Activator	Activator
Cu^2+^	5	3.12	3.48	1.58
Mn^2+^	5	3.76	Activator	Activator
Fe^2+^	5	2.83	3.96	4.43
Fe^3+^	5	0.31	2.81	2.42
EDTA	5	6.41	6.89	7.16
SDS	5	7.89	6.72	6.29
DTT	5	7.93	5.49	6.17
Triton-X100	0.2%	-	-	-
Tween-80	0.2%	-	-	-

IC_50_ represents the half inhibitory concentration. (“-”represents no enzymatic activity).

**Table 2:** Kinetic parameters of the wild-type enzyme and mutants catalyzing four kinds of substrates.

Substrates	Enzyme	*V_max_* (μM/min/mg)	*K_m_* (mM)	*K_cat_* (S^−1^)	*K_cat_*/*K_m_* (S^−1^·mM^−1^)
*p*NP-β-glu	wild-type enzyme	363.46 ± 4.13 ^c^	0.33 ± 0.03 ^c^	333.99 ± 3.80 ^c^	1012.09 ^c^
	mutant III	532.82 ± 6.51 ^b^	0.27 ± 0.05 ^b^	489.77 ± 5.98 ^b^	1813.96 ^b^
	mutant IV	841.65 ± 3.82 ^a^	0.22 ± 0.03^a^	773.64 ± 3.52 ^a^	3516.54 ^a^
*p*NP-β-gal	wild-type enzyme	230.97 ± 5.71 ^c^	0.81 ± 0.16 ^b^	212.24 ± 5.25 ^c^	262.02 ^c^
	mutant III	349.18 ± 4.83 ^a^	0.52 ± 0.17 ^a^	320.97 ± 4.44 ^a^	617.25 ^a^
	mutant IV	257.38 ± 5.47 ^b^	0.87 ± 0.24 ^c^	236.85 ± 5.03 ^b^	272.24 ^b^
*p*NP-β-xyl	wild-type enzyme	307.72 ± 3.64 ^c^	1.31 ± 0.22 ^c^	282.77 ± 3.34 ^c^	215.85 ^c^
	mutant III	337.28 ± 4.61 ^b^	1.24 ± 0.13 ^b^	310.03 ± 4.24 ^b^	250.02 ^b^
	mutant IV	358.83 ± 3.88 ^a^	1.17 ± 0.16 ^a^	330.21 ± 3.57 ^a^	282.23 ^a^
*p*NP-α-glu	wild-type enzyme	29.61 ± 1.83 ^a^	1.07 ± 0.20 ^a^	27.21 ± 1.68 ^a^	25.43 ^a^
	mutant III	25.81 ± 2.06 ^b^	1.19 ± 0.23 ^b^	23.73 ± 1.89 ^b^	19.94 ^b^
	mutant IV	19.93 ± 1.98 ^c^	1.27 ± 0.19 ^c^	18.35 ± 1.82 ^c^	14.45 ^c^

In columns, for the same substrate, means with the different lowercase superscripted letters indicate a significant difference (*p* < 0.05). Data are presented as mean ± standard deviation (n = 3).

**Table 3:** Comparison of the number of salt bridges between the wild-type enzyme and mutants.

Different Enzyme	Salt Bridge	Salt Bridge Connected to *p*-NPG
wild-type	22	-
mutant III	18	-
mutant IV	18	-
wild-type-*p*-NPG	22	0
mutant III-*p*-NPG	18	1
mutant IV-*p*-NPG	18	1

## Data Availability

The datasets presented in this article are not readily available because privacy restrictions. Requests to access the datasets should be directed to the corresponding authors.

## Supplementary Materials

The following supporting information can be downloaded at: https://www.mdpi.com/article/10.3390/ijms26093983/s1.
