# Supplementary material for: Site-Directed Mutagenesis Increased the Catalytic Activity and Stability of Oenococcus oeni β-Glucosidase: Characterization of Enzymatic Properties and Exploration of Mechanisms
Source: Int J Mol Sci. 2025 Apr 23;26(9):3983. doi: 10.3390/ijms26093983 (PMC12072002; doi:10.3390/ijms26093983)
Supplement: Supplementary file 1 [file ijms-26-03983-s001.zip › Highlights.pdf]

Highlights

- Two BGL mutants with significantly increased enzyme activity were obtained.
- Site-directed mutagenesis significantly improved the thermal stability of the BGLs.
- F133 and N181 are the key amino acids for the catalytic activity and stability.
- Hydrogen bonding and π-π interaction are main forces for enzyme catalysis.
