# Supplementary material for: Site-Directed Mutagenesis Increased the Catalytic Activity and Stability of Oenococcus oeni β-Glucosidase: Characterization of Enzymatic Properties and Exploration of Mechanisms
Source: Int J Mol Sci. 2025 Apr 23;26(9):3983. doi: 10.3390/ijms26093983 (PMC12072002; doi:10.3390/ijms26093983)
Supplement: Supplementary file 1 [file ijms-26-03983-s001.zip › Tables S1-S3.pdf]

Table S1 Mutation primer sequence

Mutational Site	Primer Sequences
Q20K	F：5'-GGCCAACAAATTAGAGGGCGGTTGGGACCAAG-3'
	R：5'-CCTCTAATTTGTTGGCCGCTACGGCGCCTCCC-3'
Q20R	F：5'-TAGCGGCCAACCGATTAGAGGGCGGTTGGGACC-3'
	R：5'-TAATCGGTTGGCCGCTACGGCGCCTCCCCACA-3'
F133K	F：5'-CGCATAAAGAAATGCCCTATCACTTGGTTAAAG-3'
	R：5'-GGGCATTTCTTTATGCGAGAGGGTAATGACCGG-3'
N181R	F：5'-GATCGACAGACAAACCGATTATACAAATCGCTTC-3'
	R：5'-CGGTTTGTCTGTCGATCTCATTAAAGGTCATCCA-3'
T244L	F：5'-GATCAACATGTTGCCTGTTTACCCGGCTTCTTCA-3'
	R：5'-CAGGCAACATGTTGATCATGCAGCCGATCTGA-3'

Table S2 Half-life of the enzymatic activity for wild-type enzyme and mutants

Enzymes	Half-life (h)
wild-type enzyme	23.00
mutant III	26.00
mutant IV	31.00

Half-life: The time it takes for enzyme activity to decrease to half of its original level.

Table S3 The prediction results of pI, Mr and Instability Factor of wild-type enzyme and mutants

Enzymes	pI	Mr（Da）	Instability Factor
wild-type enzyme	6.14	55134.47	29.57
mutant III	6.19	55152.52	28.08
mutant IV	6.19	55213.61	27.77
